# Hemophagocytic Lymphohistiocytosis in a Patient With Hodgkin’s Lymphoma Mimicking Sinusoidal Obstructive Syndrome: A Diagnostic Challenge

**DOI:** 10.7759/cureus.85397

**Published:** 2025-06-05

**Authors:** Monica Soliman, Deepa Lazarous

**Affiliations:** 1 Internal Medicine, MedStar Georgetown University Hospital, Washington, USA; 2 Critical Care Medicine, MedStar Georgetown University Hospital, Washington, USA

**Keywords:** a+avd, dacarbazine, drug-induced liver injury (dili), epstein-barr virus, hemophagocytic lymphohistiocytosis (hlh), hodgkin's lymphoma, sinusoidal obstruction syndrome

## Abstract

Hemophagocytic lymphohistiocytosis (HLH) is a rare, life-threatening hyperinflammatory syndrome triggered by malignancy, infection, or autoimmune disease. We present a case of a 19-year-old male with Hodgkin’s lymphoma who developed high-grade fevers, hepatomegaly, and severe liver injury shortly after receiving chemotherapy. He was initially diagnosed with sinusoidal obstructive syndrome (SOS) and treated with defibrotide. Despite therapy, his condition deteriorated, prompting transfer to our institution for evaluation of acute liver failure and suspected drug-induced liver injury (DILI). Further diagnostic evaluation revealed markedly elevated serum ferritin and soluble interleukin-2 receptor (sCD25), and bone marrow biopsy demonstrated prominent hemophagocytosis. These findings confirmed the diagnosis of HLH, likely secondary to underlying Hodgkin’s lymphoma and possible Epstein-Barr virus (EBV) reactivation. The patient was initiated on high-dose corticosteroids with rapid clinical and biochemical improvement. This case underscores the diagnostic complexity of HLH, particularly in lymphoma patients undergoing chemotherapy, where overlapping syndromes can delay accurate diagnosis, and highlights the critical importance of early recognition to initiate timely, life-saving immunosuppressive therapy.

## Introduction

Hemophagocytic lymphohistiocytosis (HLH) is a rare, life-threatening hyperinflammatory syndrome that results from uncontrolled immune activation and cytokine release. It is divided into primary and secondary forms. Primary HLH is usually a disease of childhood and occurs due to inherited genetic mutations that affect immune regulation [1]. On the other hand, secondary HLH mostly occurs in adulthood and may be triggered by infections, autoimmune diseases, or malignancies, particularly hematologic cancers such as Hodgkin’s lymphoma. Although the incidence of HLH is difficult to obtain due to diagnostic challenges, it has been estimated to be around one in 3,000 pediatric critical care admissions and one in 2,000 adult critical care admissions for primary and secondary HLH, respectively [1]. HLH is characterized by a constellation of non-specific findings including fever, hepatosplenomegaly, cytopenias, transaminitis, and elevated inflammatory markers, which frequently overlap with both the clinical manifestations of the underlying malignancy or infection, and the adverse effects of treatments like chemotherapy.

In patients undergoing treatment for Hodgkin’s lymphoma, this diagnostic overlap can present a significant challenge. For instance, transaminitis and persistent fever in the post-chemotherapy setting are often initially attributed to infectious etiologies or drug-induced liver injury. Dacarbazine, a commonly used alkylating agent in Hodgkin’s lymphoma regimens, has been associated with hepatotoxicity and, in rare cases, sinusoidal obstructive syndrome (SOS) [2]. These overlapping features can lead to diagnostic anchoring, delaying recognition and treatment of HLH, and increasing the risk of poor outcomes.

The consequences of delayed diagnosis are significant as HLH can rapidly lead to death. A recent systematic review reported that over 66% of patients with lymphoma-associated HLH died within a median of 5.1 months, underscoring the critical importance of early identification and intervention [3]. Here, we present a case of HLH in a 19-year-old male with Hodgkin’s lymphoma who developed acute hepatic injury following chemotherapy. Initially misdiagnosed as SOS, the patient was later found to meet diagnostic criteria for HLH. This case highlights the importance of maintaining a broad differential diagnosis and high clinical suspicion for HLH in oncology patients with unexplained systemic inflammation.

## Case presentation

A 19-year-old male with classical Hodgkin’s lymphoma, diagnosed approximately four months prior, presented to an outside hospital two days after completing his sixth cycle of chemotherapy with the A+AVD regimen (brentuximab vedotin, doxorubicin, vinblastine, and dacarbazine). He reported high-grade fevers reaching 40°C, associated with nausea and three episodes of non-bloody emesis. At the time of presentation, he had been receiving A+AVD for two months, with prior chemotherapy-related nausea and vomiting as his only notable adverse effects. Before initiating A+AVD, he was briefly treated with nivolumab, which was discontinued due to mild hepatotoxicity that resolved following a short course of corticosteroids. His past medical history was otherwise unremarkable. He was not on any daily medications, had no known drug allergies, and his family history was negative for liver disease or malignancy.

At the outside hospital where he initially presented, physical examination was notable for facial flushing, scleral icterus, and tenderness to palpation diffusely throughout his abdomen. Imaging demonstrated hepatomegaly with diffuse heterogeneous attenuation consistent with a “nutmeg liver” appearance, usually indicating congestive hepatopathy, periportal edema, hepatofugal pulsatile main portal venous flow, and elevated hepatic arterial velocity, findings suggestive of sinusoidal obstructive syndrome (SOS). Based on these findings, he was started on an infusion of defibrotide for presumed dacarbazine-induced SOS. However, this was discontinued after the patient developed adverse reactions including facial rash, flushing, tachycardia, and fever of 40.5°C. He was subsequently transferred to our hospital for further management of severe liver dysfunction.

**Figure 1:**
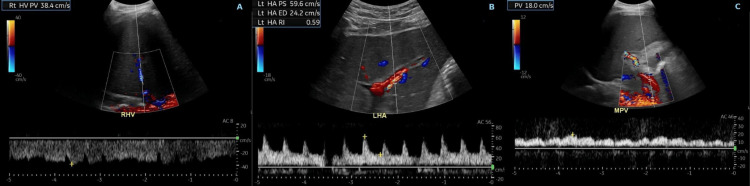
Liver ultrasound of the patient. Rt: right; HV: hepatic vein; PV: portal vein; Lt: left; HA: hepatic artery; PS: peak systolic velocity; ED: end diastolic velocity; RI: resistive index

**Figure 2:**
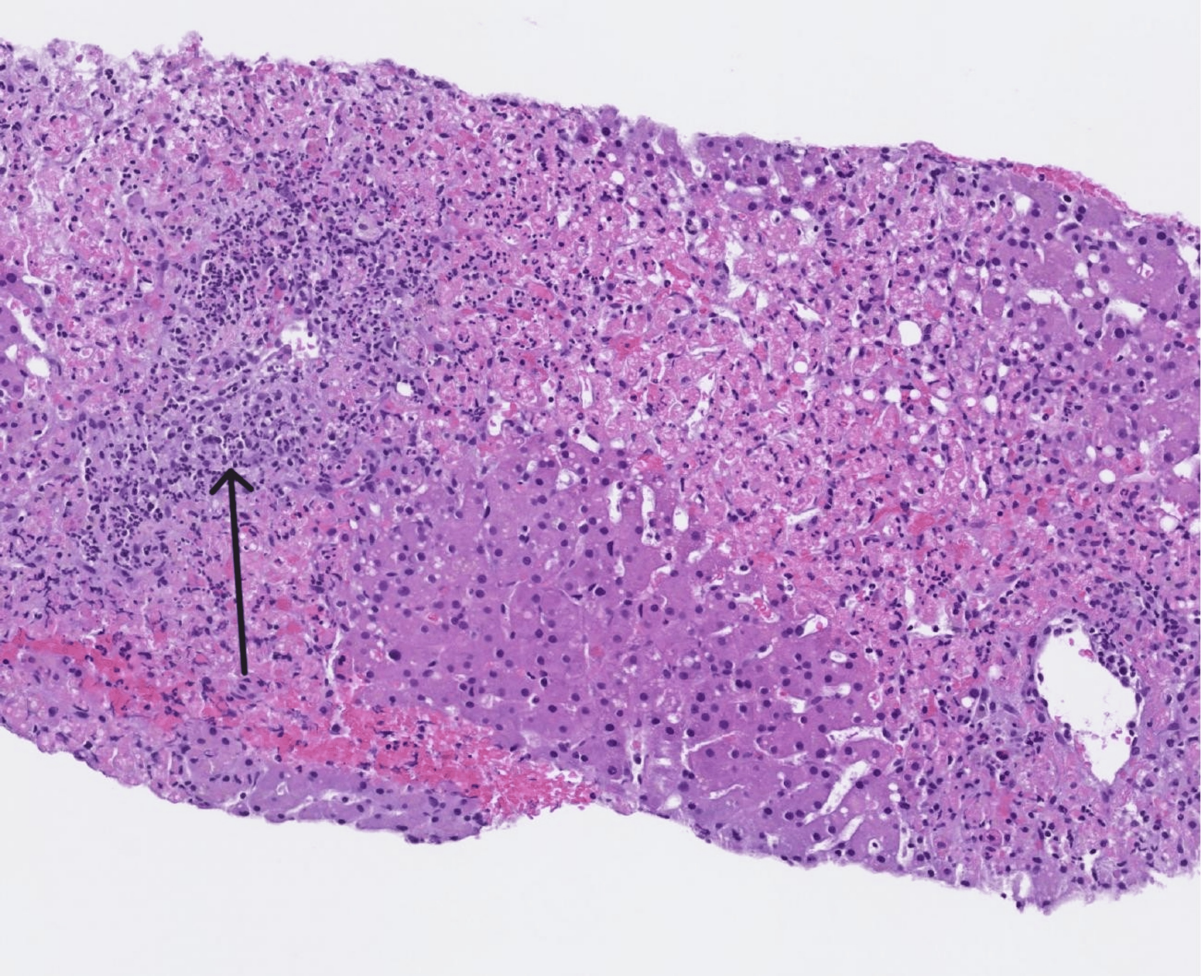
Inflammatory infiltrate and hepatic necrosis (arrow) on liver histology.

On arrival, the patient was febrile and tachycardic, with clinical signs consistent with systemic inflammatory response syndrome (SIRS). Laboratory testing revealed severe transaminitis (aspartate aminotransferase {AST}: 5,999 U/L and alanine transaminase {ALT}: 4,619 U/L) with normal alkaline phosphatase, liver synthetic dysfunction (international normalized ratio {INR}: 3.7, albumin 3.0 g/dL, platelets: 31×10⁹/L), elevated lactate (6.5 mmol/L), leukocytosis (WBC: 18×10⁹/L), and anion gap metabolic acidosis. Serum ethanol and acetaminophen levels were undetectable. Both Epstein-Barr virus (EBV) DNA and IgG levels were elevated. A broad infectious workup, including blood and urine cultures and chest radiography, was performed, all of which returned negative. However, given leukocytosis and anion gap lactic acidosis, the patient was placed on broad-spectrum antibiotics including cefepime and vancomycin, but unfortunately, continued to worsen. A repeat liver ultrasound duplex at our institution revealed an enlarged liver with normal morphology and surface contour. Hepatic vasculature was patent, with preserved direction of blood flow, which was discordant with imaging from the outside hospital performed just one day prior (Figures 1A-1C). Given this discrepancy and persistently worsening liver enzymes, a liver biopsy was pursued. Histopathology demonstrated extensive hepatic necrosis with a mixed inflammatory infiltrate predominantly composed of neutrophils (Figure 2). There was no evidence of viral inclusions, sinusoidal obstruction, or lymphoma involvement. These findings effectively ruled out SOS, raising concern for drug-induced liver injury (DILI) and acute liver failure. The patient was initiated on N-acetylcysteine infusion. 

**Figure 3:**
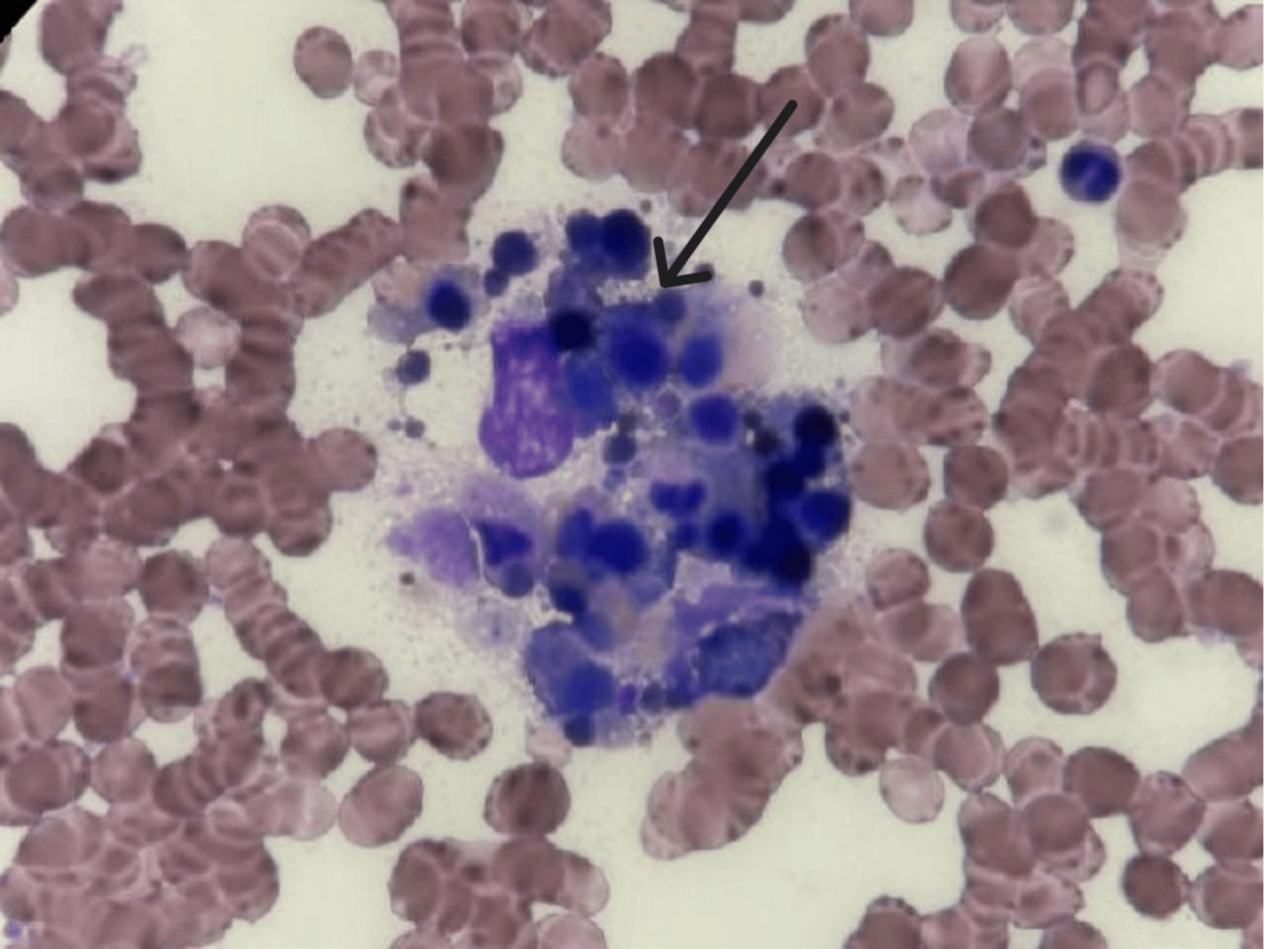
Hemophagocytosis (arrow) on bone marrow histology.

In light of persistent fever, leukocytosis, and systemic inflammation, serum ferritin was measured and found to be markedly elevated at 16,500 ng/mL. A soluble interleukin-2 receptor (sCD25) level was also obtained and returned significantly elevated at 6,822 pg/mL. His calculated HScore was 204, corresponding to an 88-93% probability of hemophagocytic lymphohistiocytosis (HLH). A bone marrow biopsy demonstrated a hypercellular marrow (90%) with myeloid predominance and prominent hemophagocytosis, confirming the diagnosis (Figure 3). The patient was promptly initiated on dexamethasone 20 mg daily, which led to clinical stabilization and improvement in liver function. He was eventually discharged on a tapering course of oral corticosteroids with close outpatient hematology follow-up. At the time of discharge, laboratory values had significantly improved, including platelets 244 ×10⁹/L, AST 105 U/L, ALT 1,198 U/L, and ferritin 2517.9 ng/mL (Table 1).

**Table 1:** Laboratory values summary. AST: aspartate aminotransferase; ALT: alanine transaminase; INR: international normalized ratio; EBV: Epstein-Barr virus

Laboratory test	On presentation	On discharge	Reference range
AST (U/L)	5,999	105	10-33
ALT (U/L)	4,619	1,198	10-49
Alkaline phosphatase (U/L)	96	98	44-147
Total bilirubin (mg/dL)	3.4	2.0	0.3-1.2
INR	3.7	1.2	0.8-1.2
Albumin (g/dL)	3.0	4.8	3.5-5.0
WBC (×10⁹/L)	18	4.4	4-11.0
Platelets (×10⁹/L)	31	244	150-450
CO_2_ (mmol/L)	14	25	20-31
Lactate (mmol/L)	6.5	1.0	0.5-2.2
Anion gap (mmol/L)	16	11	5-15
Triglycerides (mg/dL)	179	74	0-149
Ferritin (ng/mL)	16,500	2517.9	10-307
Acetaminophen (µ/mL)	7	Not repeated	10-30
Ethanol (mg/dL)	<3	Not repeated	<3
EBV IgG (units/mL)	>750	Not repeated	0-21
EBV DNA PCR (copies/mL)	228	Not repeated	<200
sCD25 (pg/mL)	6,822	Not repeated	175-858

## Discussion

This report illustrates the diagnostic complexity of hemophagocytic lymphohistiocytosis (HLH) in the setting of Hodgkin's lymphoma and potential chemotherapy-induced liver injury. Differentiating HLH from other chemotherapy-related complications, such as sinusoidal obstructive syndrome (SOS) and drug-induced liver injury (DILI), is essential, as management strategies differ significantly. While SOS and DILI are largely supportive or organ-protective in treatment, HLH requires prompt immunosuppressive therapy - typically corticosteroids and sometimes etoposide - to prevent life-threatening complications [4].

Sinusoidal obstructive syndrome is most commonly associated with myeloablative chemotherapy regimens used prior to hematopoietic stem cell transplantation (HSCT), particularly in allogeneic HSCT settings. It rarely occurs outside of this context [5]. In this case, the initial diagnosis of SOS was likely based on classic radiologic findings and hepatic dysfunction; however, the absence of HSCT and eventual biopsy findings made this diagnosis less likely. It results from inflammatory injury to sinusoidal endothelial cells, leading to necrosis and obstruction of small hepatic venules [5]. Clinically, this manifests as painful hepatomegaly, jaundice, ascites, and laboratory abnormalities such as elevated aminotransferases (up to 8000 U/L), thrombocytopenia, and hyperbilirubinemia [6]. While defibrotide remains the primary treatment, severe or refractory cases have been managed with high-dose corticosteroids and, in rare instances, liver transplantation [5].

In contrast, HLH is a hyperinflammatory syndrome marked by unregulated immune activation. It can be triggered by infection, autoimmune disease, or malignancy. Malignancy-associated HLH, especially in patients with hematologic cancers, is well-documented. Interestingly, there also seems to be a strong association between Epstein-Barr virus (EBV) positivity and HLH in patients with Hodgkin's lymphoma [7]. This relationship may suggest a synergistic immunologic trigger in lymphoma patients with concurrent EBV reactivation.

The role of chemotherapy in precipitating HLH remains unclear. While chemotherapeutic agents are immunosuppressive by design, certain regimens may paradoxically act as triggers in susceptible individuals. There are case reports describing HLH in the context of Hodgkin's lymphoma treated with the A+AVD regimen (brentuximab vedotin, doxorubicin, vinblastine, dacarbazine). One report described a 63-year-old man who achieved complete remission of both HLH and lymphoma after six cycles of A+AVD [8], while another described a 26-year-old patient who developed HLH during treatment with brentuximab vedotin and nivolumab and ultimately died [9]. The authors noted the possibility that HLH was an adverse event occurring after administration of this chemotherapy regimen [9]. These conflicting outcomes underscore the need for more research into the relationship between specific chemotherapeutic agents and the development of HLH.

The clinical diagnosis of HLH is based on a combination of clinical, laboratory, and histopathologic criteria. The HLH-2004 diagnostic guidelines require five or more of the following: fever, splenomegaly, cytopenias affecting at least two lineages, hypertriglyceridemia, hyperferritinemia, hemophagocytosis on tissue biopsy, and elevated soluble interleukin-2 receptor (sCD25) [4]. Our patient met all of these criteria. The HScore, a validated scoring system for HLH probability in adults, can also guide diagnosis, with our patient’s score of 204 indicating an 88-93% likelihood of HLH. Although bone marrow biopsy is not required to confirm the diagnosis, it is often performed to evaluate for other causes of cytopenias and may reveal characteristic findings of hemophagocytosis, as it did in our patient. Management of HLH depends on severity; in some cases, treating the underlying cause (e.g., infection or malignancy) may be sufficient. In more severe cases, immunosuppressive therapy following the HLH-94 protocol - consisting of dexamethasone and etoposide, with intrathecal methotrexate or hydrocortisone for central nervous system involvement - is warranted [4]. While our patient only required steroids and had significant improvement after a few days of his hospitalization, the biggest limitation of this case is that we were unable to monitor his clinical status following hospital discharge, as he eventually established care at an outside facility.

## Conclusions

Hemophagocytic lymphohistiocytosis (HLH) should remain a critical consideration in the differential diagnosis of patients presenting with persistent fever, cytopenias, and systemic inflammation, particularly in the context of underlying malignancy or autoimmune disease. Early recognition and prompt initiation of immunosuppressive therapy are essential to reducing morbidity and mortality. This case underscores the importance of maintaining a high index of suspicion for HLH, even when alternative diagnoses such as sinusoidal obstructive syndrome or drug-induced liver injury appear plausible. Multidisciplinary collaboration is vital in navigating complex clinical presentations involving overlapping inflammatory syndromes and rapidly evolving liver dysfunction.

## References

[1] Konkol S, Killeen RB, Rai M (2025). Hemophagocytic lymphohistiocytosis. StatPearls [Internet].

[2] (2012). Antineoplastic agents. LiverTox [Internet].

[3] Knauft J, Schenk T, Ernst T, Schnetzke U, Hochhaus A, La Rosée P, Birndt S (2024). Lymphoma-associated hemophagocytic lymphohistiocytosis (LA-HLH): a scoping review unveils clinical and diagnostic patterns of a lymphoma subgroup with poor prognosis. Leukemia.

[4] Henter JI, Horne A, Aricó M (2007). HLH-2004: diagnostic and therapeutic guidelines for hemophagocytic lymphohistiocytosis. Pediatr Blood Cancer.

[5] Fleming S, Scott AP, Coutsouvelis J (2024). ANZTCT practice statement: sinusoidal obstruction syndrome/veno-occlusive disease diagnosis and management. Intern Med J.

[6] Venkatesh SK, Harper KC, Borhani AA (2024). Hepatic sinusoidal disorders. Radiographics.

[7] Jin Z, Wang Y, Wei N, Wang Z (2020). Hodgkin lymphoma-associated hemophagocytic lymphohistiocytosis - a dangerous disease. Ann Hematol.

[8] Knox B, Singh D, Mai H, Mirza K (2019). Hodgkin's lymphoma with HLH and complete remission with brentuximab-based therapy. BMJ Case Rep.

[9] Mosalem O, Pai T, Alqawasma M, Shaikh M, Li KD, Alhaj Moustafa M (2024). Severe cytokine release syndrome and hemophagocytic lymphohistiocytosis (HLH)-like syndrome following administration of combined brentuximab vedotin and nivolumab for recurrent classical Hodgkin lymphoma: a case report. J Blood Med.

